# Transfer learning model for anomalous event recognition in big video data

**DOI:** 10.1038/s41598-024-78414-2

**Published:** 2024-11-13

**Authors:** Roqaia Adel Taha, Aliaa Abdel-Halim Youssif, Mohamed Mostafa Fouad

**Affiliations:** College of Computing and Information Technology, Arab Academy for Science, Technology and Maritime Transport (AASTMT), Smart Village, Cairo, Egypt

**Keywords:** Computational biology and bioinformatics, Mathematics and computing, Physics

## Abstract

Video surveillance faces challenges due to the need for improved anomalous event recognition techniques for human activity recognition. Growing security concerns make standard CCTV systems insufficient because of high monitoring costs and operator exhaustion. Therefore, automated security systems with real-time event recognition are essential. This research introduces a semantic key frame extraction algorithm based on action recognition to minimize frame volume big video data. This approach has not been previously applied with ResNet50, VGG19, EfficientNetB7, and ViT_b16 models for recognizing anomalous events in surveillance videos. The findings demonstrate the effectiveness of this method in achieving high accuracy rates. The proposed method addresses the challenges posed by large volumes of frames generated by surveillance videos, requiring effective processing techniques. A large number of videos from the UCF-Crime dataset were used for proposed model evaluation, including both abnormal and normal videos during the training and testing phase. EfficientNetB7 achieved 86.34% accuracy, VGG19 reached 87.90%, ResNet50 attained 90.46%, and ViT_b16 excelled with 95.87% accuracy. Compared to state-of-the-art models from other studies, the transformer model (ViT_b16) outperformed these algorithms, demonstrating significant improvements in recognizing anomalous events.

## Introduction

Video surveillance played a crucial role in law enforcement, transportation, and environmental monitoring. It was increasingly essential in fields such as traffic control, criminal prevention, and investigation. However, accurate, real-time recognition of anomalous events from videos posed ongoing challenges in the fields of machine learning and computer vision. The vast amount of real-time streams produced by surveillance systems exceeded current capacities. Manual monitoring of surveillance cameras 24/7 was challenging and costly, as studies had shown that CCTV system operators’ attention spans significantly decreased after 20 minutes of monitoring^1^.

Automated video content analysis, including the recognition of anomalous events, could benefit various surveillance applications. However, the lack of temporal annotations in large datasets made anomalous event detection and recognition challenging tasks. Most researchers viewed anomalous event recognition as a distinct problem that required training models^2,3^. Several techniques, such as expanded Kalman filter, hybrid support vector machine, stacked auto-encoder, and histogram of gradients (HOG), had been employed to analyze traffic accidents and identify anomalous events^4,5^. However, the success of these algorithms in classifying other anomalous events had been limited, suggesting restrictions in their application to surveillance contexts.

Deep Learning (DL) techniques had shown impressive results in computer vision applications and time series analyses. They were efficient for analyzing video sources and had achieved state-of-the-art outcomes for anomalous event detection-related issues^2,5^. Convolutional neural networks (CNNs) were a subset of DL neural networks specifically engineered to process visual data, including image and video recognition. Various CNN designs, such as ResNet-50, EfficientNetB7, Inception-v3, and VGG19, had been presented to solve the problem of anomalous events recognition in videos^2,6–8^. CNN models like ResNet50, VGG19, and EfficientNetB7 were utilized in this study. Furthermore, a deep learning model called Vision Transformer was proposed, which achieved the highest accuracy among all the models trained.

In this research, semantic key frame extraction algorithm applied to a large number of videos was focused on to recognize anomalous events in big video data. This study aimed for a model that could differentiate between various forms of violence, going beyond traditional anomalous event detection. Anomalous event recognition was complicated due to the many different violent action types that could have similarities even if they belonged to separate classes. The use of the most recent Vision Transformer architecture for feature extraction utilizing transfer learning and semantic key frame extraction algorithm was utilized to tackle these problems.

The key contributions of this paper were summarized as follows:

- This research introduces a a Semantic Key Frame Extraction Algorithm for extracting key frames based on action recognition. The algorithm reduces the volume of video frames, addressing the challenges posed by processing large amounts of data in surveillance videos.
- The study explores the use of advanced deep learning models, specifically ResNet50, VGG19, EfficientNetB7, and the Vision Transformer (ViT_b16), for the first time in the context of recognizing anomalous events in surveillance videos. These models are applied using the extracted frames from the proposed semantic key frame extraction algorithm.
- The proposed approach demonstrates significant improvements in the accuracy of anomalous event detection, with ViT b16 achieving 95.87% accuracy, outperforming other state-of-the-art models.
- The research employs the UCF-Crime dataset, a large and diverse dataset containing both normal and abnormal video instances, providing a robust evaluation of the model’s performance in real-time scenarios.
- By focusing on real-time event recognition, this research addresses the growing need for automated security systems to reduce the costs and limitations associated with human monitoring in video surveillance.

A summary of relevant work on anomalous event recognition methods is provided in “Related work” section. The proposed methods for identifying anomalous events are presented in “Proposed methods” section, which also introduces the dataset, the spatially semantic key frame extraction algorithm, and models fine-tuning. The appropriate experimental results are provided in “Results” section. Finally, the Conclusion is provided in “Conclusions” section accordingly.

## Related work

In this section, the crucial role of anomalous event recognition in surveillance videos was highlighted in various applications such as security, public safety, and anomalous event detection. Significant research efforts have been devoted to developing effective algorithms and models for detecting anomalous events using datasets like the UCF-Crime dataset. In this paper, some of the most recent studies in this domain were reviewed, and their methodologies and results were discussed. Anomalous event recognition in surveillance videos has been an active area of research, with recent studies focusing on leveraging the UCF-Crime dataset for evaluation and benchmarking purposes. In this section, some of the most recent papers in this domain were reviewed.

### Anomalous event detection in videos using deep learning

Sultani, Chen, and Shah^9^ introduced the UCF-Crime dataset, which encompassed a wide range of violence classifications (14 types). The DMIL Ranking model was proposed, treating anomalous event detection as a regression problem within a multiple instance learning (MIL) framework. Their approach employed a ranking-based loss function to train a fully connected neural network for anomalous event detection. The results of several recent deep learning baselines on anomalous activity recognition were provided, achieving 75.41%. Anala, Makker, and Ashok^10^ developed a system capable of identifying unusual behavior in surveillance videos by utilizing three frames per second instead of one, enabling the capture of subtle changes. However, their evaluation was limited to normal videos, indicating potential limitations in solely relying on normal data for anomalous event recognition. They utilized 3DCNN for feature extraction on the UCF-Crime dataset. They extracted spatial features, and Long Short-Term Memory (LSTM) networks learned the sequences. The classification, using a CNN-LSTM model, achieved an accuracy of 85%. Hao et al.^11^ proposed a two-stream convolutional network model for anomalous event detection by combining RGB and Flow networks. They treated anomalous event detection as a regression problem and utilized a CNN-Residual network (ResNet) for feature extraction on the UCF-Crime dataset. They achieved an accuracy of 81.22%. Venkatesh et al.^12^ discussed a crime detection method suitable for deep learning-based on-device monitoring to reduce delay, data gathering costs, and privacy violations. They achieved an accuracy of 80.23%. Their approach utilized Early-Stopping-MIL and Long Short-Term Memory (LSTM) concepts for anomalous event recognition on the UCF-Crime dataset. Ullah et al.^13^ presented a lightweight CNN-based anomalous event recognition system for surveillance with reduced time complexity. They utilized pre-trained lightweight CNN-multilayer and BiLSTM on the UCF-Crime dataset for anomalous event detection. They achieved an accuracy of 78.43%. Wu et al.^14^ introduced a CNN and multiple instance learning (MIL) approach, employing a pre-trained VGG16 model for feature extraction and Gaussian background modeling for moving target extraction on the UCSD dataset. The multiple instance learning can accurately locate the abnormal events in this work. Boekhoudt et al.^15^ created the Human connected crime (HR-crime) subset of the UCF-Crime dataset for recognizing anomalous events connected to humans. They developed a feature extraction pipeline and analyzed HR-crime baseline outlier recognition. They achieved an accuracy of 60.30%. Cao et al.^16^ proposed an Adaptive Graph Convolutional Network (GCN) for video anomalous event detection, accounting for both temporal differences and feature similarity when building a global graph. Their method employed an adaptive graph learning layer to capture spatial-temporal correlations between video segments.

In video-based action recognition, frame redundancy and minimal inter-frame differences can hinder deep network learning. Many approaches used key frame extraction algorithms or random frame sampling strategies to address this challenge, although most learnable frame selection techniques lacked independence and were tailored for specific models.

### Anomalous event recognition in videos using transfer learning

Wang et al.^17^ introduced a unsupervised anomalous event detection method based on spatiotemporal autoencoders. They demonstrated competitive results on the UCF-Crime dataset, outperforming traditional supervised approaches.

Liu et al.^18^ presented a graph convolutional network (GCN) architecture for anomalous event detection in surveillance videos. They achieved promising results on the UCF-Crime dataset, leveraging both spatial and temporal information.

### Action recognition using key frame extraction

The massive volume of videos created and posted online every day had generated a growing need for more effective frame selection techniques. The primary goal of these methods was to choose the most important frames from videos without leaving out any key frames. Several techniques had been suggested for frame selection^19–22^. These techniques either employed the frame-based selection strategy^21^ or the segment-based selection approach^22^. Another frame-based sampler, called Attention-Aware Sampling (AAS)^23^, chose important frames by using REINFORCE to train an agent and produce importance ratings that the attention module used for action recognition. A segment-based salient sampler called SCSampler^22^ could extract useful segments from lengthy, untrimmed videos. It included samples of multiple audiovisual segments from videos, each of which had a saliency score assigned to it. The sampler was trained to rate the importance score of each video based on the derived classification features.

### Action recognition using vision transformer

By utilizing the potential of the Transformer^24^, ViT^25^ overcame logical disadvantages such as translation invariance and spatially restricted receptive fields in images. To achieve this, it divided an image into a series of patches, flattened them, created linear embeddings, added positional embeddings to determine the location of each patch in the original image, and fed this sequence as input to a standard transformer encoder (comprised of a multi-head attention layer that enabled the model to concurrently attend to information from various representation parts at different positions)^24^. The achievements of Transformers, ViT, and their variants^26^ were undeniable, including object detection in videos^27^, action recognition^28^, and frame synthesis^29^. The method utilized here was distinct from earlier suggestions, as it was based on key frame extraction algorithm^30^ and ViT^25^, compared to other deep learning models. This was the first instance of anomalous event recognition using this key frame extraction algorithm for anomalous event recognition^30^.

**Fig. 1:**
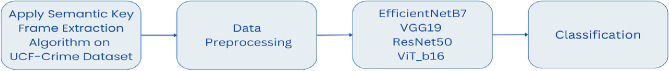
The general architecture of the proposed model.

## Proposed methods

**Fig. 2:**
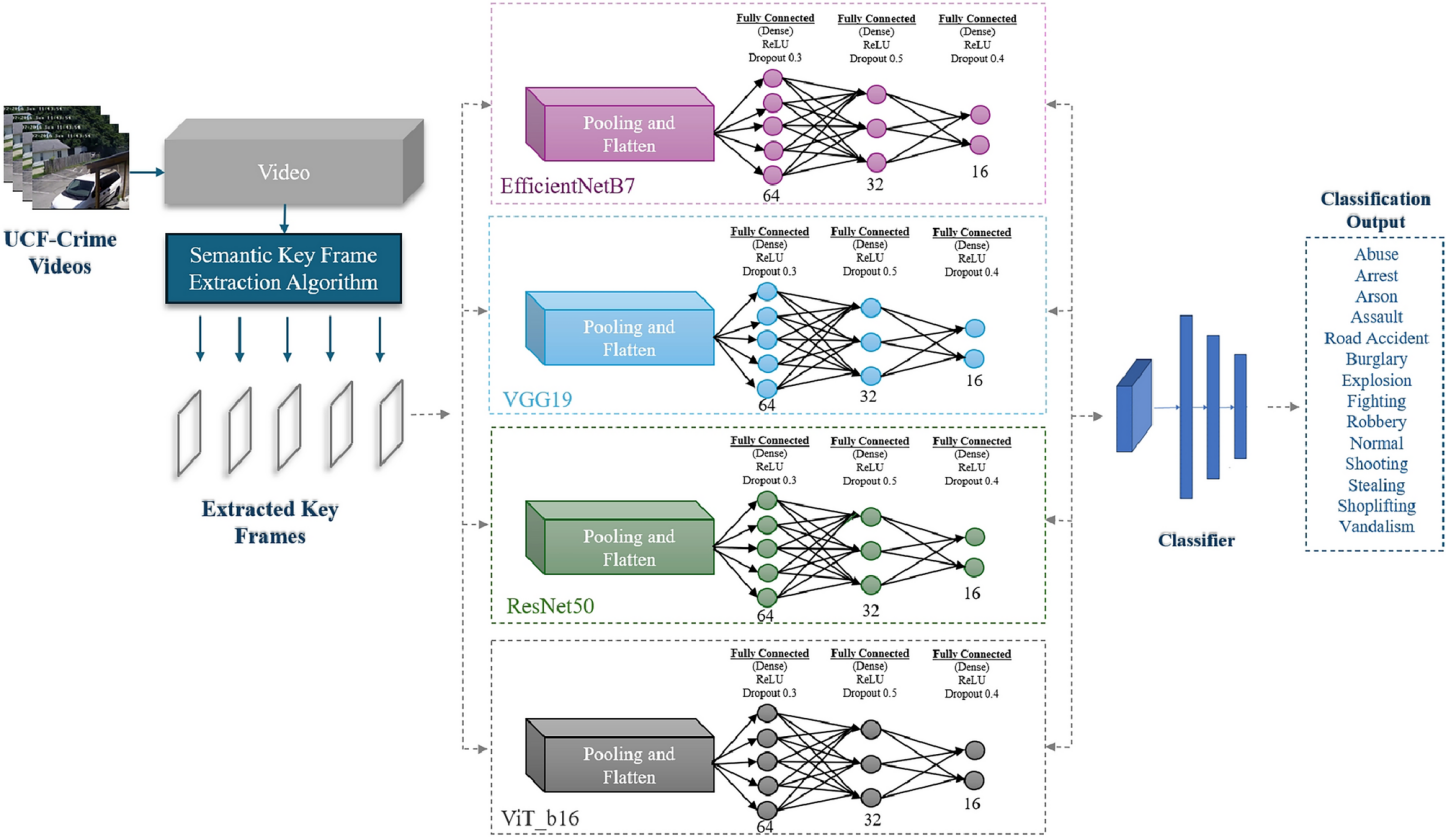
The detailed proposed model architecture for anomalous event recognition using the UCF-Crime dataset. Frames are extracted from videos using semantic key frame extraction algorithm.

This section present the model and architecture that were utilized to solve the problem. The general picture of the proposed model is shown in Fig. 1 .Along with the changes made for the use case, an in-depth description of the model was given. A model was put proposed in two distinct phases. Initially, DFPicker, a semantic key frame extraction technique, was introduced^30^. Second, to extract features from the input data, ResNet50, VGG19, EfficientNetB7, and ViT_b16 were used for both pretraining and fine-tuning. In addition, a classifier tuning was created, as shown in Fig. 2, which makes it easier to compare several models in terms of semantic key frame extraction. In order overcome surveillance big video data problems, the semantic key frame extraction algorithm (applied on 1600 videos from the UCF-Crime dataset) and transfer learning were utilized to work with the latest Vision Transformer deep architecture and three additional deep learning models for feature extraction.

### Dataset

A standard dataset for research on the recognition of criminal activities was the UCF-Crime dataset. Developed by the University of Central Florida (UCF)^31^, it was freely accessible for usage in research projects by academics and professionals. The videos in the dataset varied in quality and resolution and were gathered from a variety of sources, including YouTube, TV news, and documentaries. It consisted of 1900 uncut surveillance videos, totaling 128 hours of footage. Thirteen actual criminal incidents were shown in the recordings, including Abuse, Arrest, Arson, Assault, Road Accident, Burglary, Explosion, Fighting, Robbery, Shooting, Stealing, Shoplifting, and Vandalism. The substantial influence these activities had on public safety led to their selection. The average video length was 7.2 seconds, with a frame rate of 25 and 30 FPS. The UCF-Crime dataset also presented a number of difficulties, including changes in the foreground’s scale, crowded backdrops, and viewpoint and motion modifications of the camera. Moreover, a few of the videos included fluctuations in brightness, noise, and poor quality. Apart from the aforementioned difficulties, the UCF-Crime dataset exhibited fluctuations in terms of the quantity of individuals engaged in the activities, their clothes, and the kinds of weapons employed, among other aspects. These differences posed challenges for creating reliable and precise criminal activity recognition systems. The suggested approach aimed to identify every class in the UCF-Crime dataset for the particular study. To do this, the semantic key frame extraction algorithm^30^ was used to extract the frames, which were then divided into two overlapped training and validation splits: 20% for testing (75,716 frames) and 80% for training (302,866 frames). There was a total of 378,582 frames.

### Semantic key frame extraction

Specifically in the context of the UCF-Crime dataset, the integration of the semantic key frame extraction algorithm with anomalous event recognition provided a way to improve video analysis for surveillance and security applications.

Many methods, including object detection, activity recognition, and semantic segmentation, were used to choose the key frames. These methods made it possible to extract useful visual data by assisting in the identification of frames that included relevant information about criminal activity.

**Fig. 3:**
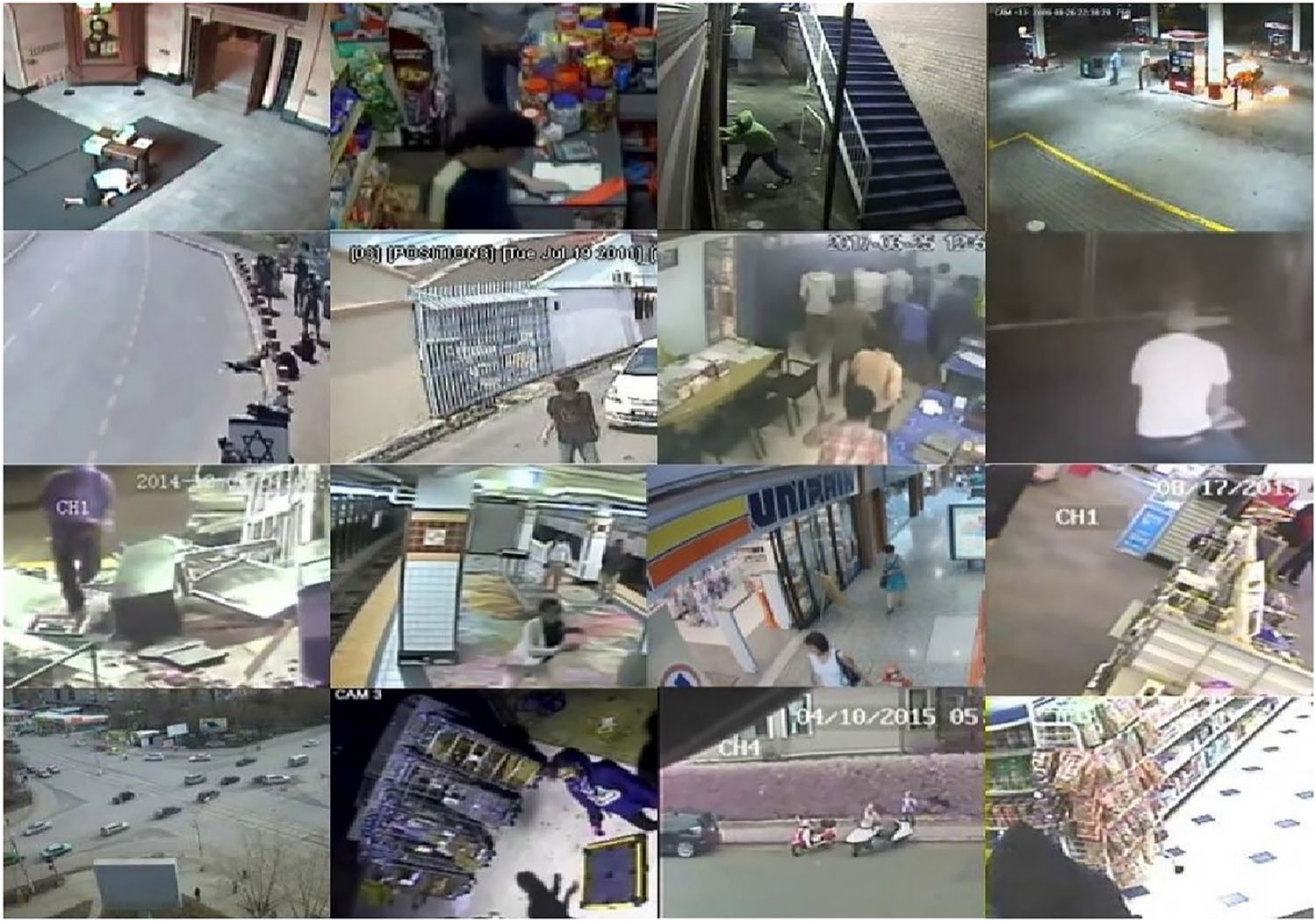
Extracted key frames samples from UCF-Crime dataset.

A deeper recognition of the context and content of the surveillance event was achieved by the model through the integration of the semantic key frame extraction algorithm with anomalous event recognition as described in Algorithm 1. This made it possible to identify and categorize anomalous events in the UCF-Crime dataset like explosions, abuse, and vandalism more precisely. The key frames samples that extracted from UCF-Crime dataset are shown in Fig. 3.

Discriminative Frames Picker (DFPicker)^30^ was a proposed video semantic key frame extraction algorithm that utilized many crucial processes to select representative frames for action recognition. At first, a preset temporal interval was used to divide the video into temporal non-overlapping sliding windows. Every sliding window was made up of consecutive frames, the length and frame rate of the video determined how many windows there were and how big they were. A temporal stride and dilation were added to improve flexibility, allowing frames to be skipped when calculating motion. The motion of each sliding window’s frame pairs was then scored using either pixel-wise absolute difference or sparse optical flow. The highest-scoring frame pairs which were chosen to represent important action moments in the video were subject to a motion measure criteria. The motion computation process was designed to operate in real-time, and scalability and efficiency were made possible by the GPU-based implementation. Furthermore, by processing frame pairs independently and adaptably, various streams allowed the model to dynamically adapt to different sliding window sizes. With all factors considered, DFPicker provided an adjustable and scalable method of frame selection, improving the effectiveness of video summary for tasks involving action recognition. More information is provided on the motion computation techniques, Motion with Optical Flow and Motion with Pixel-Wise Absolute Difference. These approaches will be discussed in more detail in the following. **Motion with Optical Flow.** For two key reasons, the algorithm used sparse optical flow to compute the optical flow rather than dense optical flow: first, because neighboring pixels typically had very similar motions, the object intra-pixel intensities did not change substantially between consecutive frames. Second, sparse optical flow was employed to accelerate the selection process and demonstrated comparable performance in comparison to the pixel-wise absolute difference method. By using this approach, the selection process might be completed more quickly and in real-time. If there was minimal difference in the frame brightness of two consecutive frames, the velocity $V$ of the optical flow was obtained by simplifying $rf$, $V = f$ to $AV = b$ with the least squares method resulting in $A^T AV = A^T b$, which corresponded to $V = (A^T A)^{-1} A^T b$. Where:

- $V$ represented the optical flow velocity.
- $A$ was a matrix representing the relationship between the optical flow velocity and the displacement of frame intensities.
- $b$ represented the displacement of frame intensities.

So, the expression $AV = b$ represented the relationship between the optical flow velocity and the displacement of frame intensities.

**Motion with pixel-wise absolute difference**. More complicated methods like optical flow were needed for motion computation in order to track objects and estimate depth. For video summary, on the other hand, the pixel-wise absolute difference demonstrated competitive performance because the summarization algorithm tried to remove frame redundancy as in Eq. (1). As a result, one option in the procedure was to compute the motion score for pixel-wise motion computation $d_{ab}$ between the frames $f$ and $f'$ as:

1 $$\begin{aligned} \begin{aligned} d =&\sum _{i=1}^{h} \sum _{j=1}^{w} \Vert f(i,j) - f'(i,j) \Vert , \\&\text {to obtain a set of scores } d \in \mathbb {R} \text { for each sliding window } C_z. \end{aligned} \end{aligned}$$

### Data pre-processing and augmentation

The following data preprocessing procedures were carried out in this study:

- Gathering and Labeling Data: A variety of data from the anomalous event recognition dataset UCF-Crime was collected from the Kaggle dataset. Various anomalous event types, such as abuse, assault, arson, robbery, explosion, road accident, fighting, shooting, stealing, shoplifting, and vandalism, were categorized based on frames extracted by a semantic key frame extraction algorithm.
- Data Splitting: The dataset was separated into training and testing sets. This allowed for the evaluation of the model’s robustness and performance across various data subsets.
- Image Resizing: All images were scaled to 256x256 pixels, and a constant size of 64x64 for models like ResNet50, EfficientNetB7, and VGG19. This ensured homogeneity in input dimensions.
- Color Mode: All images were kept in RGB color mode to adhere to the standard format frequently used in deep learning projects. This modification made it easier to employ categorical cross-entropy loss during model training.
- Data Augmentation: Data augmentation approaches were employed during training to increase the model’s robustness and capacity to generalize. Augmentation techniques included rescaling, flipping, and random rotations. By artificially increasing the diversity of the training data, data augmentation facilitated better model learning.

**Fig. 4:**
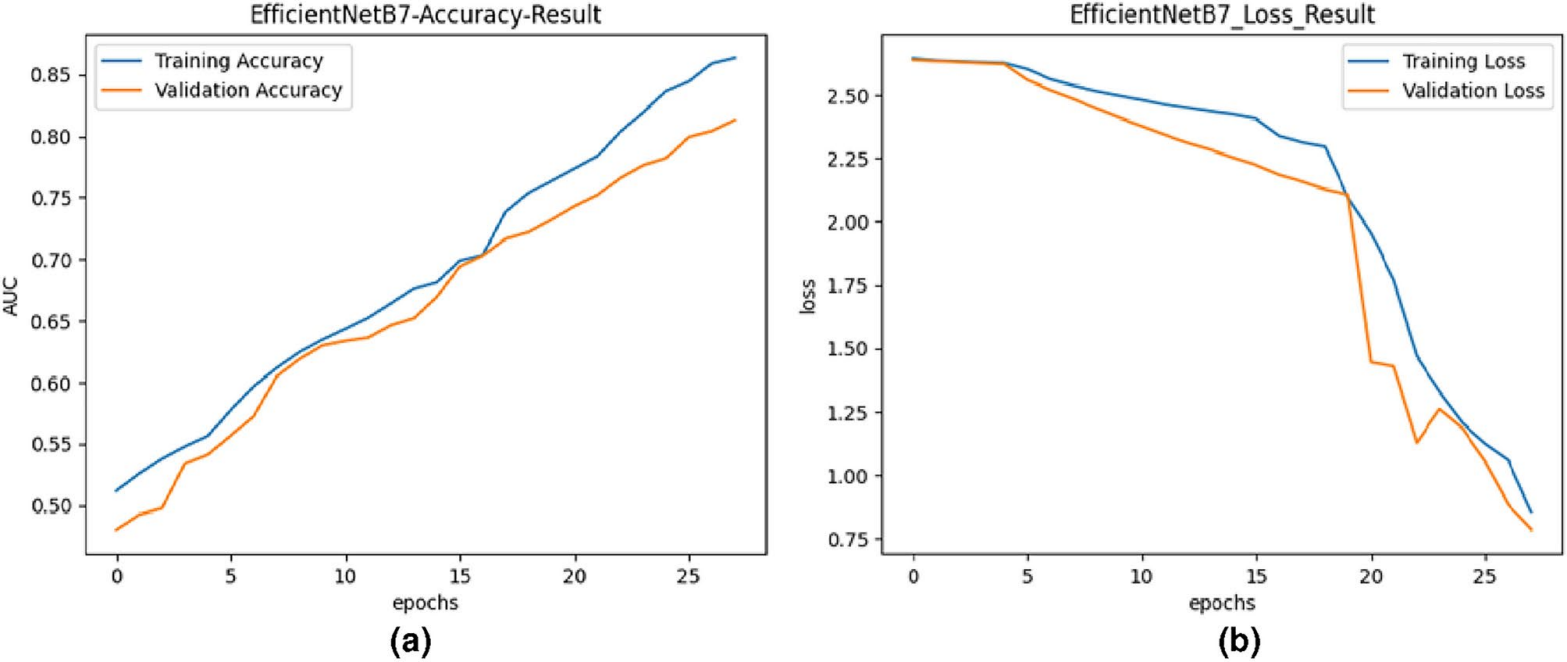
Training and validation curves for EfficientNetB7.

**Fig. 5:**
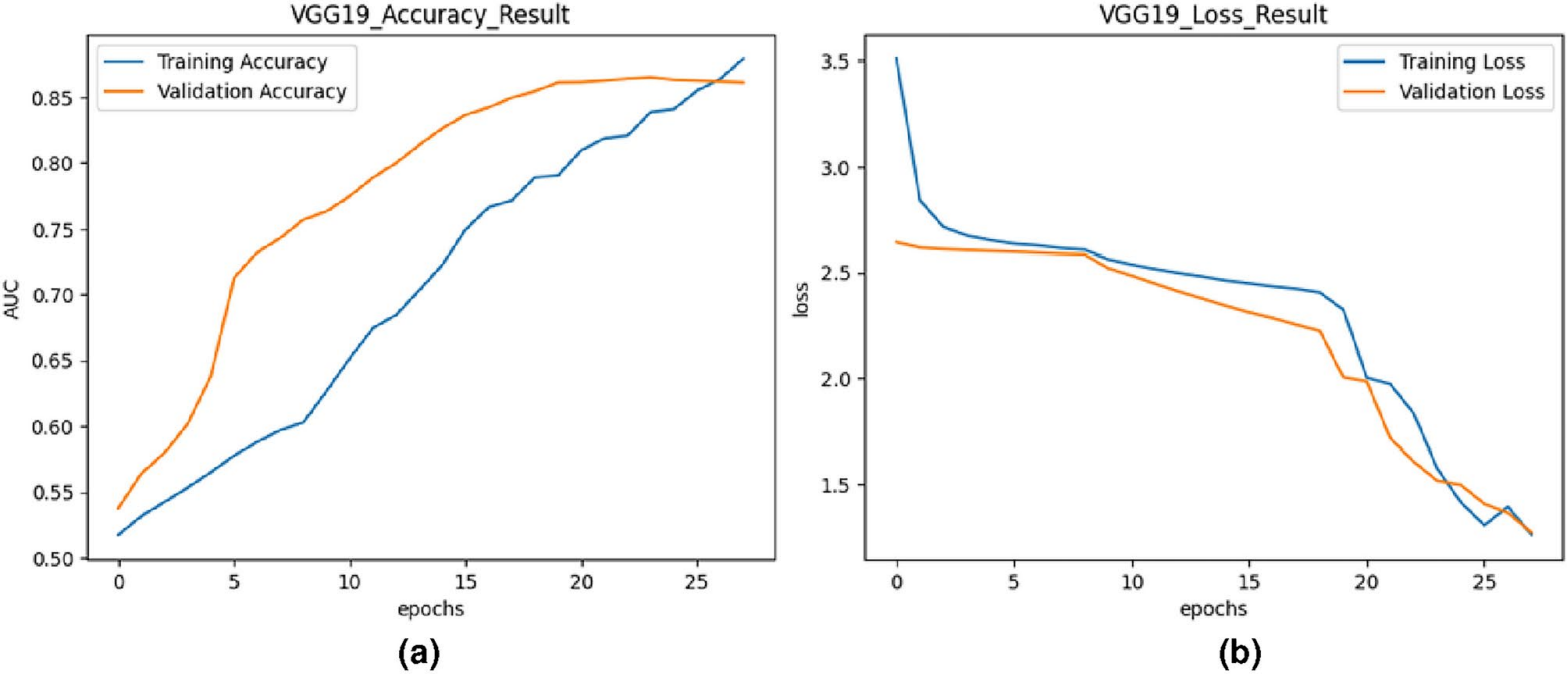
Training and validation curves for VGG19.

**Fig. 6:**
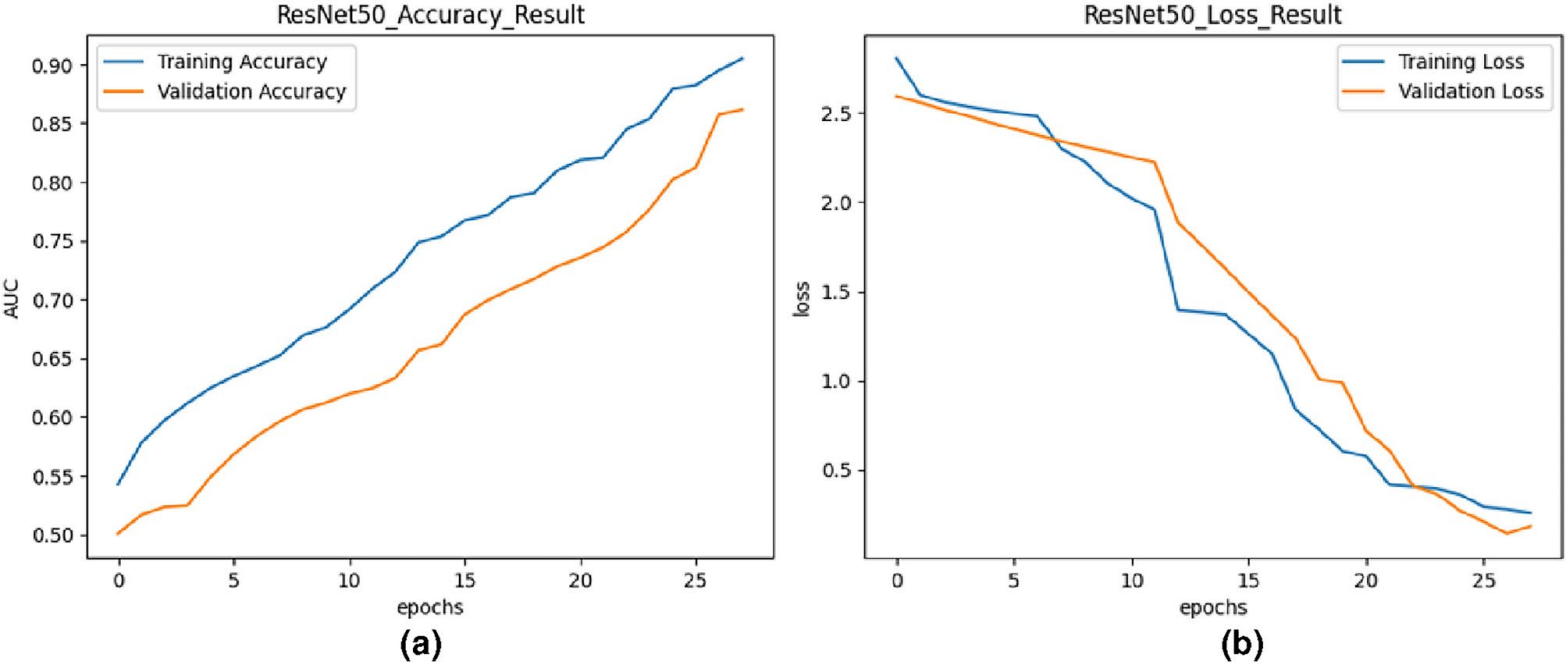
Training and validation curves for ResNet50.

**Fig. 7:**
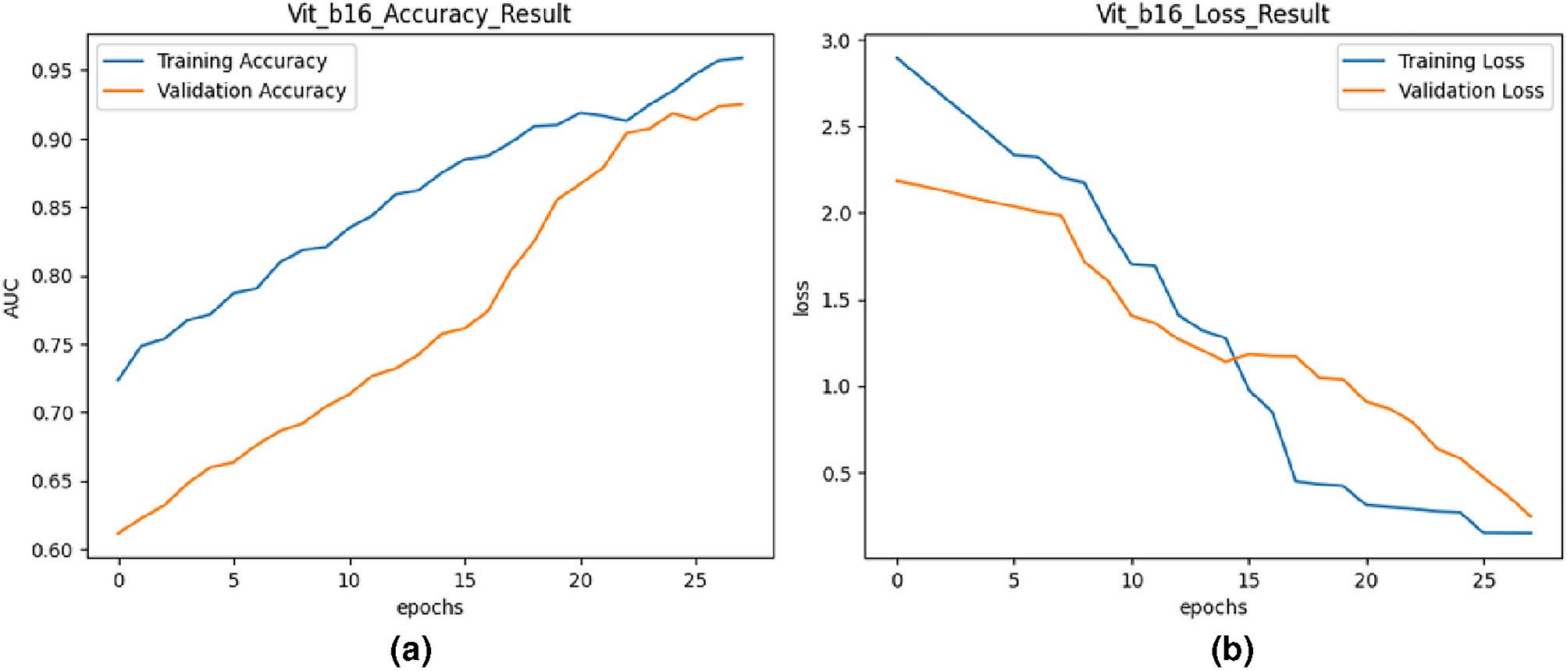
Training and validation curves for ViT_b16.

### The proposed framework

In the proposed model, state-of-the-art architectures, namely ResNet50, VGG19, EfficientNetB7, and Vision Transformer (ViT_b16), are employed to enhance the recognition of anomalous events in video surveillance as described in Algorithm 1. ResNet50 utilizes skip connections to ease the training of deep networks, where the output of a block is defined in Eq. (2):

2 $$\begin{aligned} Y = F(X) + X \end{aligned}$$

In this equation, $Y$ is the output of the residual block, $F(X)$ represents the transformation applied to the input $X$, allowing for improved gradient flow during training. VGG19 is built on sequential convolutional layers followed by pooling, with the convolution operation expressed in Eq. (3):

3 $$\begin{aligned} Y = \text {ReLU}(W *X + b) \end{aligned}$$

where $Y$ is the output of the convolutional layer, $W$ is the weight tensor for the convolution operation, $X$ is the input to the layer, and $b$ is the bias term. The $\text {ReLU}$ function applies a non-linear activation to introduce non-linearity in the model.

EfficientNetB7 leverages depthwise separable convolutions and compound scaling for efficiency, represented in Eq. (4):

4 $$\begin{aligned} Y = \text {DepthwiseConv}(X) *W_{\text {pointwise}} + b \end{aligned}$$

where $Y$ is the output of the depthwise separable convolution, $\text {DepthwiseConv}(X)$ represents the depthwise convolution applied to the input $X$, $W_{\text {pointwise}}$ is the weight matrix for the pointwise convolution, and $b$ is the bias term.

**Fig. 8:**
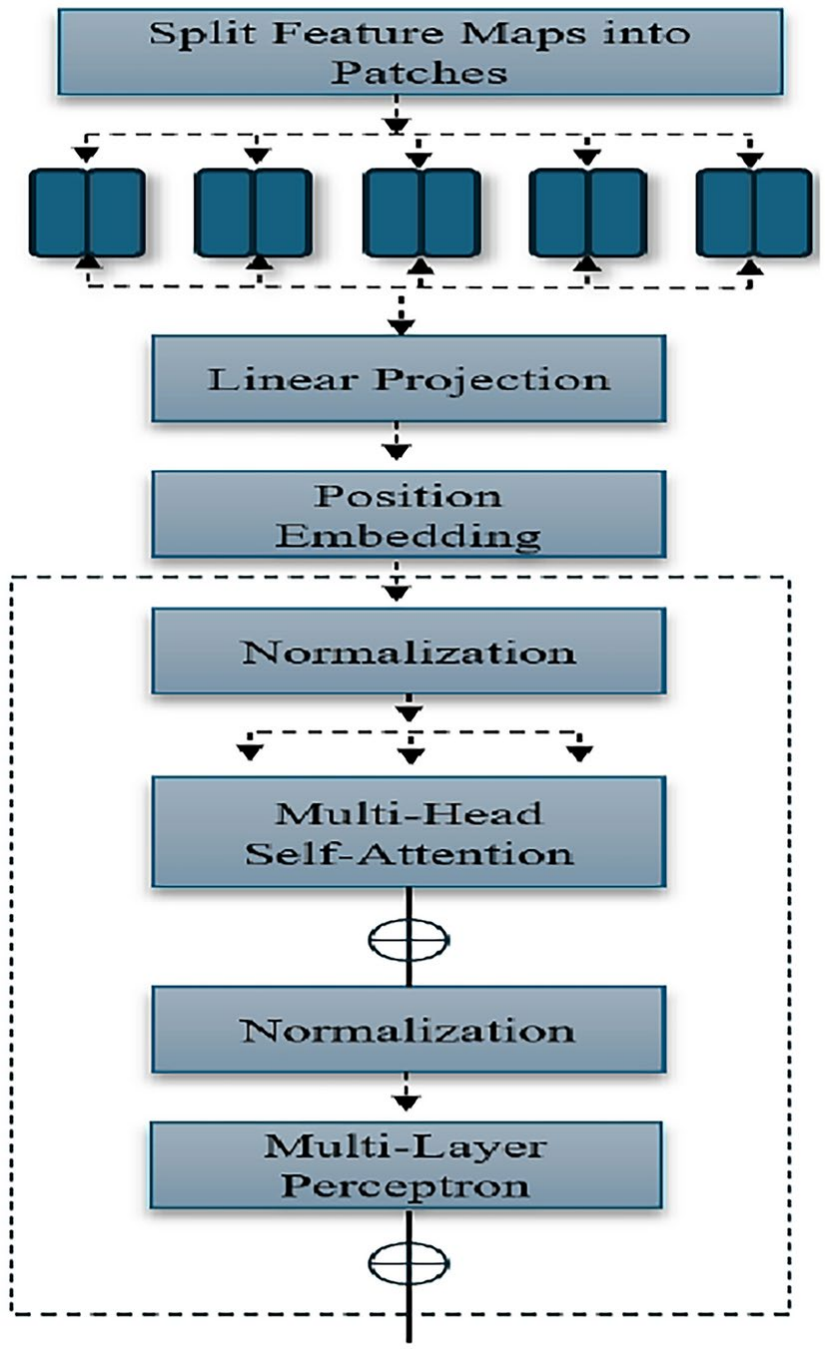
Vit_b16 architecture.

ViT_b16 is focused on patch embeddings and self-attention, where the input image $X$ is divided into patches that are flattened and projected into an embedding space, as shown in Eq. (5):

5 $$\begin{aligned} Z = \text {Flatten}(X_{\text {patches}}) \cdot W_p + b_p \end{aligned}$$

where $Z$ represents the flattened patch embeddings, $X_{\text {patches}}$ is the input image divided into patches as shown in Fig. 8, $W_p$ is the weight matrix for the projection, and $b_p$ is the bias term added to the projections. This allows for efficient representation of spatial information. Positional encodings are added in Eq. (6):

6 $$\begin{aligned} Z' = Z + E_{\text {pos}} \end{aligned}$$

where $Z'$ is the updated patch embedding, and $E_{\text {pos}}$ represents the positional encoding that provides information about the position of each patch in the input image. Self-attention mechanisms are utilized to enable the model to focus on relevant parts of the input, as expressed in Eq. (7):

7 $$\begin{aligned} Z_{l+1} = \text {LayerNorm}(Z_l + \text {MLP}(\text {SelfAttention}(Z_l))) \end{aligned}$$

where $Z_{l+1}$ is the output of the self-attention layer at the $(l+1)$-th layer, $Z_l$ is the input to this layer, and $\text {MLP}$ represents a multi-layer perceptron. The $\text {LayerNorm}$ function normalizes the input to improve training stability.

**Algorithm 1:**
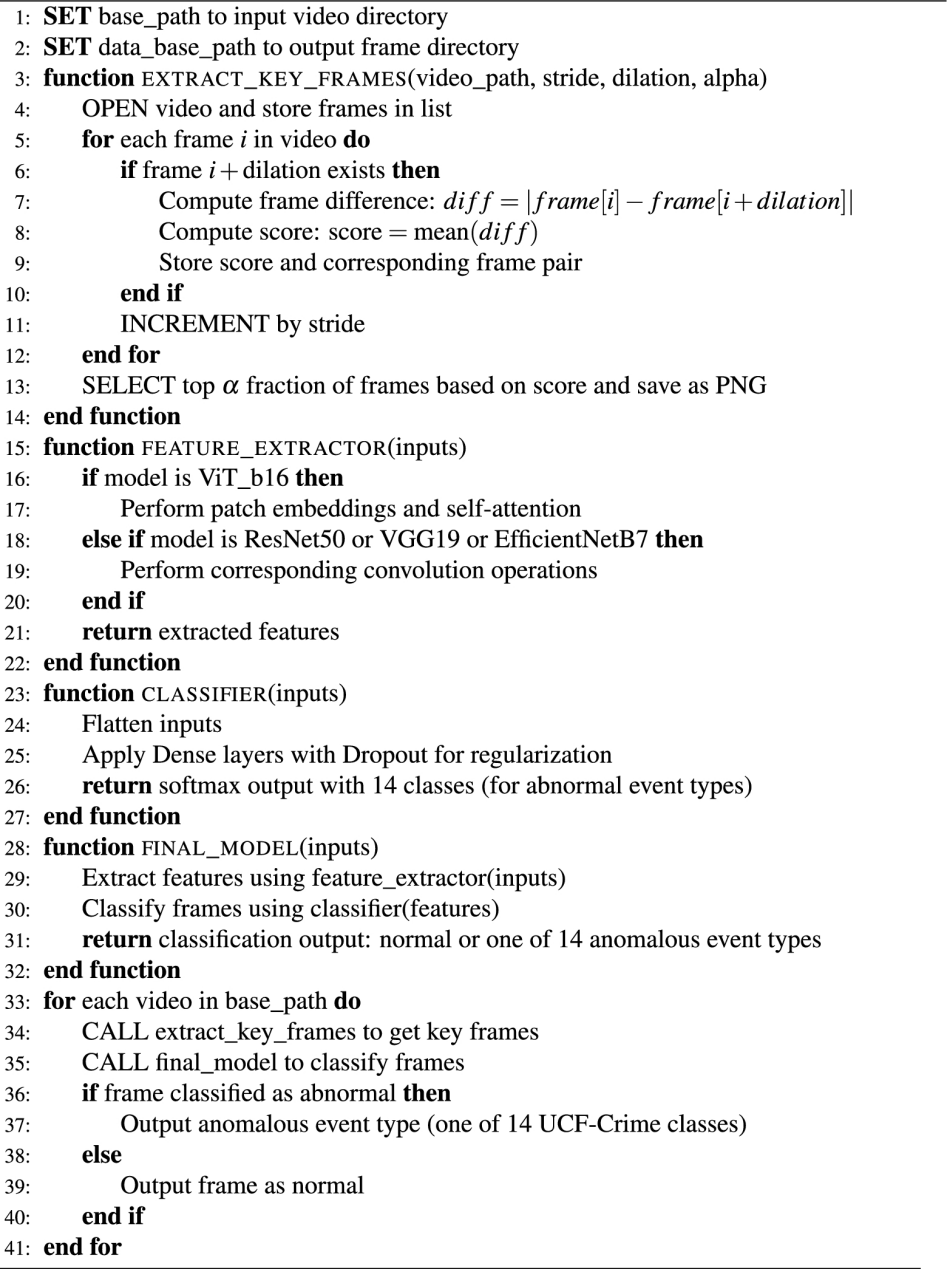
Semantic key frame extraction and event classification

### Hyper-parameters tuning

The present study involve the fine-tuning of pre-trained models through weight adjustments in order to maximize their performance for the recognition of anomalous events in video surveillance. The goal was to adjust the weights of models like ViT_b16, ResNet50, VGG19, EfficientNetB7, and others to the specifics of the target dataset while making use of the information gained during their initial training. Pre-trained weights were loaded, suitable loss functions and optimizers were defined, and the models were trained on the target dataset. The objective was to improve the models’ capacity to accurately detect and classify anomalous events in surveillance videos by fine-tuning, thus raising the system’s overall efficiency. The hyperparameters employed in this study are listed in Table 1.

**Table 1:** Hyper-parameters for proposed models.

Hyperparameter	Value
Learning rate	0.00003
Batch size	64
Optimizer	SGD
Dropout rate	0.3
Activation functions	RELU

### Classification module

The weights of the feature vectors were trained by the feature classification module to identify various types of anomalous events, such as Abuse, Arrest, Arson, Assault, Road Accident, Burglary, Explosion, Fighting, Robbery, Shooting, Stealing, Shoplifting, and Vandalism. The following layers were included in this module:

- A global average pooling layer: was applied to produce a fixed-length feature vector by reducing the spatial dimensions of the input feature maps.
- A flatten layer: was used to create a 2048-feature vector from the output of the preceding layer, which was then supplied into a fully connected layer.
- A dense (fully connected) layer: consisted of a ReLU activation function and 1024 neurons. Every neuron had connections to 2048 feature vector nodes.
- A dropout layer: was applied to reduce overfitting, enhance the model’s resistance to noise, and help it adapt to new data. With a dropout rate of 0.3, 30% of the neurons were lost at random while being trained.
- A Softmax layer: Final predictions are made using this layer, which uses the deep features extracted from the frames sequence to identify whether the sequence consists of normal or anomalous events that pass through the models.

**Table 2:** Results of the proposed models.

Model	Training accuracy	Validation accuracy	Training loss	Validation loss
EfficientNetB7	0.86	0.81	0.85	0.78
VGG19	0.87	0.86	1.25	1.26
ResNet50	0.90	0.86	0.25	0.18
ViT_b16	0.95	0.92	0.14	0.24

## Results

The studies discussed in this section highlight the recent advancements in anomalous event recognition using the UCF-Crime dataset. These approaches leverage various techniques including deep learning, unsupervised learning, graph-based methods, attention mechanisms, and meta-learning techniques, contributing to the development of more effective surveillance systems. Four state-of-the-art convolutional neural network architectures, namely EfficientNetB7, VGG19, ResNet50, and ViT_b16, were trained and evaluated. Each model was trained using the same dataset and experimental setup.

### Training and validation accuracy and loss

To further analyze the performance of each model, including transfer learning frameworks, the training and validation accuracy and loss curves were presented. These curves provided insights into the convergence behavior and generalization performance of the models. In addition to the models trained using transfer learning frameworks, the performance of a semantic key frame extraction algorithm for anomalous event recognition in surveillance videos was also evaluated. Semantic key frame extraction algorithm involved identifying and selecting frames that contained meaningful visual information relevant to the anomalous event recognition task.

#### The proposed model based on EfficientNetB7

EfficientNetB7 is a convolutional neural network architecture known for its efficiency and effectiveness in image classification tasks. In this study, transfer learning was employed with EfficientNetB7, leveraging pre-trained weights on a large-scale image dataset. This approach allowed the model to be adapted to the anomalous event recognition task using the UCF-Crime dataset. Figure 4 illustrates the training and validation accuracy and loss curves for the EfficientNetB7 model. The training accuracy steadily increased during training, reaching a plateau after 25 epochs. The validation accuracy followed a similar pattern, indicating good generalization performance. The training and validation loss decreased continuously.

#### The proposed model based on VGG19

VGG19 is a deep convolutional neural network architecture with 19 layers, known for its simplicity and effectiveness in various computer vision tasks. In this study, transfer learning was adopted with VGG19, initializing the model with weights pre-trained on large-scale image datasets. The model was fine-tuned on the UCF-Crime dataset for anomalous event recognition. Figure 5 depicts the training and validation accuracy and loss curves for the VGG19 model. The training and validation accuracy curves showed a similar trend to Transfer Learning - EfficientNetB7, with both metrics increasing over epochs. However, the validation accuracy was slightly lower than the training accuracy, indicating a slight degree of overfitting. The validation loss changed but was generally regular, while the training loss reduced very quickly.

#### The proposed model based on ResNet50

ResNet50 is a deep residual neural network architecture known for its ability to train very deep networks effectively. In this study, transfer learning was utilized with ResNet50, leveraging pre-trained weights on large-scale image datasets. The model was fine-tuned on the UCF-Crime dataset for anomalous event recognition. Figure 6 displays the training and validation accuracy and loss curves for the ResNet50 model. The training and validation accuracy curves exhibited a similar trend as Transfer Learning - VGG19, with both metrics increasing over epochs. The training and validation loss decreased continually.

#### The proposed model based on ViT_b16

Vision Transformer (ViT) is a transformer-based model architecture originally designed for image classification tasks. In this study, transfer learning was employed with ViT_b16, utilizing pre-trained weights on large-scale image datasets. The model was fine-tuned on the UCF-Crime dataset for anomalous event recognition. Figure 7 showcases the training and validation accuracy and loss curves for the ViT_b16 model. These curves provided insights into the training dynamics and generalization performance of each model, aiding in model selection and performance comparison. The training and validation accuracy curves demonstrated consistent improvement over epochs, with the validation accuracy closely tracking the training accuracy. Both training and validation loss decreased steadily throughout training, indicating effective convergence and generalization. The results of the semantic key frame extraction algorithm, along with the transfer learning models, were summarized in Table 2. It was observed that while the semantic key frame extraction algorithm achieved reasonable accuracy and loss values.

### Vit_b16 transfer learning framework performance

There are various reasons behind the ViT_b16 model’s better performance when it comes to anomalous event recognition with the UCF-Crime dataset. Vision transformers contain self-attention, which enables them to recognize both local and global relationships among image patches. This is in contrast to convolutional neural networks (CNNs), which are restricted by fixed receptive fields. Better performance may result from this, particularly when working on jobs that require capturing fine-grained features throughout an image or long-range dependencies. ViT_b16’s architecture made it possible for it to efficiently extract temporal and spatial data from surveillance videos, which was essential for correctly identifying anomalous events. Because ViT_b16 was pre-trained on a sizable dataset of varied images, like ImageNet,

**Table 3:** Comparison with other methods on UCF-Crime.

Year - Author	Methods used	AUC/Accuracy (%)
2018 - Kuldeep Biradar^32^	VGG19	72.66
2021 - Wu et al.^33^	Fully connected layers	84.89
2021 - Jia-Chang Feng^33^	Self-guided attention boosted feature encoder	81.55
2022 - Joshi^34^	ResNet50	75.00
2022 - Hitesh Sapkota^35^	Multiple-instance learning (MIL)	80.87
2022 - Shuo Li, Fang Liu^36^	Transformer-based with Multi-Sequence Learning	82.85
2023 - Aqib Mumtaz^37^	Inflated Inception network (I3D)	83.70
2024 - Anas Al-lahham^3^	ID3 (Iterative Dichotomiser 3)	80.65
2024 - Ahmed Elmetwally^38^	ResNet50	82.85
2024 - Hamza Karim^39^	ViT	85.92
**EfficientNetB7 + Key Frame Extraction (Proposed)**		**86.34**
**VGG19 + Key Frame Extraction (Proposed)**		**87.90**
**ResNet50 + Key Frame Extraction (Proposed)**		**90.46**
**ViT_b16 + Key Frame Extraction (Proposed)**		**95.87**

Bold values represent the proposed models utilized in this study.

it was able to acquire general features that could be adjusted for the particular purpose of anomalous event recognition. ViT_b16’s architecture and training process may have given it robustness to changes in viewpoints, brightness, and other elements seen in video surveillance. Its ability to generalize well to new data was made possible by this robustness, which improved its ability to identify unusual events. To enhance performance on the UCF-Crime dataset, several hyperparameters of ViT_b16, such as learning rate, batch size, and optimizer settings, were meticulously adjusted. Optimizing these hyperparameters was essential to getting the best results from deep learning models.

### Comparison with the proposed models

In this section, the performance of various video anomalous event recognition methods proposed by different researchers was compared. Table 3 summarized the AUC or accuracy results of each approach, along with the model used. The comparison included both traditional methods and recent deep learning-based approaches. The methodologies analyzed included a range of techniques from fully connected layers to deep learning architectures such as self-attention encoders and transformer-based models. Each approach demonstrated varying levels of effectiveness in recognizing anomalous events within video data. In recent years, there had been a major rise in the development of deep learning models tailored for video anomalous event recognition. Notable contributions included the utilization of hierarchical feature representations, graph convolutional networks, CNN, and multi-instance learning techniques. These advancements aimed to enhance the model’s ability to capture spatial-temporal dependencies and semantic features crucial for anomalous recognition tasks. The proposed models, including EfficientNetB7, VGG19, ResNet50, and ViT_b16, augmented with semantic key frame extraction algorithm, represented the point of this study. By leveraging semantic key frame extraction algorithm, these models achieved improvements in accuracy compared to existing methodologies. The UCF-Crime dataset served as the primary benchmark for evaluating the performance of these models, offering a diverse range of real-time scenarios for anomalous recognition tasks. The evaluation framework involved training and evaluating the proposed models on the UCF-Crime dataset, a large-scale public-domain dataset specifically designed for video anomalous event recognition. Through extensive experimentation, it was demonstrated that the proposed models achieved competitive results in terms of accuracy, outperforming existing methodologies in various aspects. Furthermore, the scalability of these models was demonstrated through their ability to handle big video data, which is essential for real-time surveillance applications. The efficient processing and analysis of large volumes of video data are critical for timely and accurate anomalous recognition in complex environments. By demonstrating robust performance on the UCF-Crime dataset, which contains a substantial amount of video data, the proposed models showcase their potential for deployment in real-time surveillance systems operating at scale (Fig. 8).

## Conclusion

In this study, a Semantic Key Frame Extraction Algorithm was introduced to reduce video frame volumes for more efficient processing of large-scale video datasets in surveillance applications. Advanced deep learning models, including ResNet50, VGG19, EfficientNetB7, and the Vision Transformer (ViT_b16), were applied to recognize anomalous events in surveillance videos. The results showed that EfficientNetB7 achieved 86.34% accuracy, VGG19 reached 87.90%, ResNet50 attained 90.46%, and ViT_b16 excelled with the highest accuracy of 95.87%. These findings demonstrate that the ViT_b16 model outperformed state-of-the-art approaches on the UCF-Crime dataset. This work addresses the growing demand for automated and real-time security systems by tackling key challenges in video surveillance, such as operator fatigue and the high cost of continuous monitoring. The use of transfer learning shows the potential for scalable, high-accuracy anomalous recognition in video streams. While promising results have been achieved, several areas for further research remain. One area involves improving the scalability of the model, ensuring better generalization to larger and more diverse datasets beyond UCF-Crime. Another focus should be the real-time integration of the method into live surveillance systems to enable immediate detection and response to anomalous events. Additionally, ensemble learning techniques could be explored to combine the strengths of multiple models, potentially enhancing accuracy and robustness. Adapting the model to different environmental conditions (e.g., lighting changes, weather variations) in surveillance footage would increase its applicability in real-world scenarios. Investigating methods to further reduce computational costs without sacrificing accuracy would also be valuable for deploying the system in resource-limited environments.

## Supplementary Information

Supplementary Information.

## Data Availability

The data utilized in this study, the Anomaly Detection Dataset UCF, is publicly available on Kaggle, https://www.kaggle.com/datasets/minhajuddinmeraj/anomalydetectiondatasetucf. Additionally, the extracted key frames from the semantic key frame extraction algorithm used in this study are publicly available at the following link: https://drive.google.com/drive/folders/1FHxGa8Vcq86mN26KzuGCKzJa7RM5GdNx?usp=sharing.

## Code availability

The dataset used in this study, including the extracted frames, is publicly available at the following link: https://drive.google.com/drive/folders/1FHxGa8Vcq86mN26KzuGCKzJa7RM5GdNx?usp=sharing. The Anomaly Detection Dataset UCF is also publicly available on Kaggle: https://www.kaggle.com/datasets/minhajuddinmeraj/anomalydetectiondatasetucf.
