# Supplementary material for: Transfer learning model for anomalous event recognition in big video data
Source: Sci Rep. 2024 Nov 13;14:27868. doi: 10.1038/s41598-024-78414-2 (PMC11560924; doi:10.1038/s41598-024-78414-2)
Supplement: Supplementary file 1 — Supplementary Information. [file 41598_2024_78414_MOESM1_ESM.pdf]

Supplementary Information

Title of the Manuscript

Transfer Learning Model for Anomalous Event Recognition in Big Video Data

Authors

Roqaia Adel Mohammed
College of Computing and Information Technology,
Arab Academy for Science, Technology and Maritime Transport (AASTMT),
Smart Village, Cairo, Egypt
Email: rokaiaadel2020@gmail.com

Aliaa Abdel-Halim Youssif
College of Computing and Information Technology,
Arab Academy for Science, Technology and Maritime Transport (AASTMT),
Smart Village, Cairo, Egypt
Email: aliaay@aast.edu

Mohamed Mostafa Fouad
College of Computing and Information Technology,
Arab Academy for Science, Technology and Maritime Transport (AASTMT),
Smart Village, Cairo, Egypt
Email: mohamed.mostafa@aast.edu

Figures

Figure 1

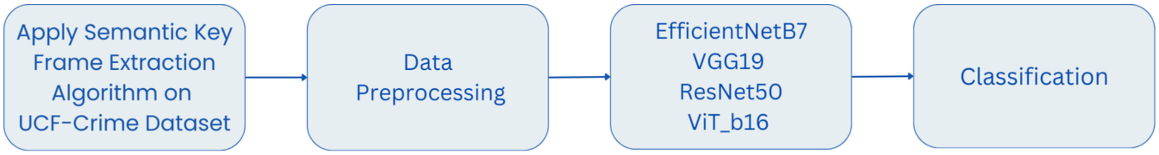

Figure 1 **The general architecture of the proposed model.**

Figure 2

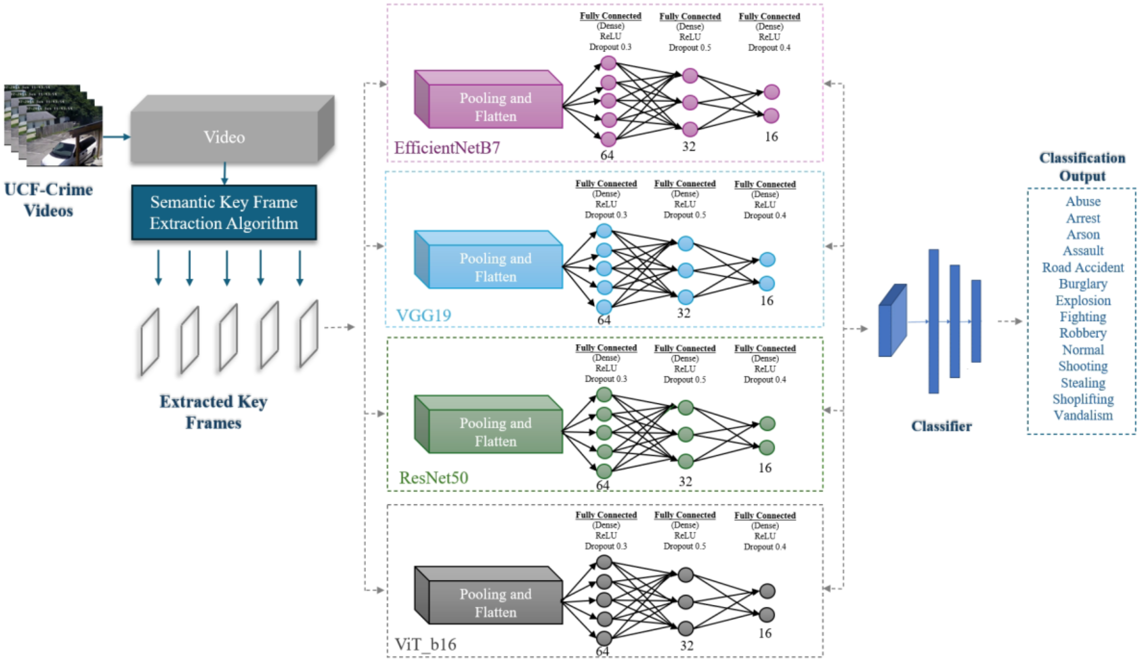

Figure 2 **The detailed proposed model architecture**

Figure 3

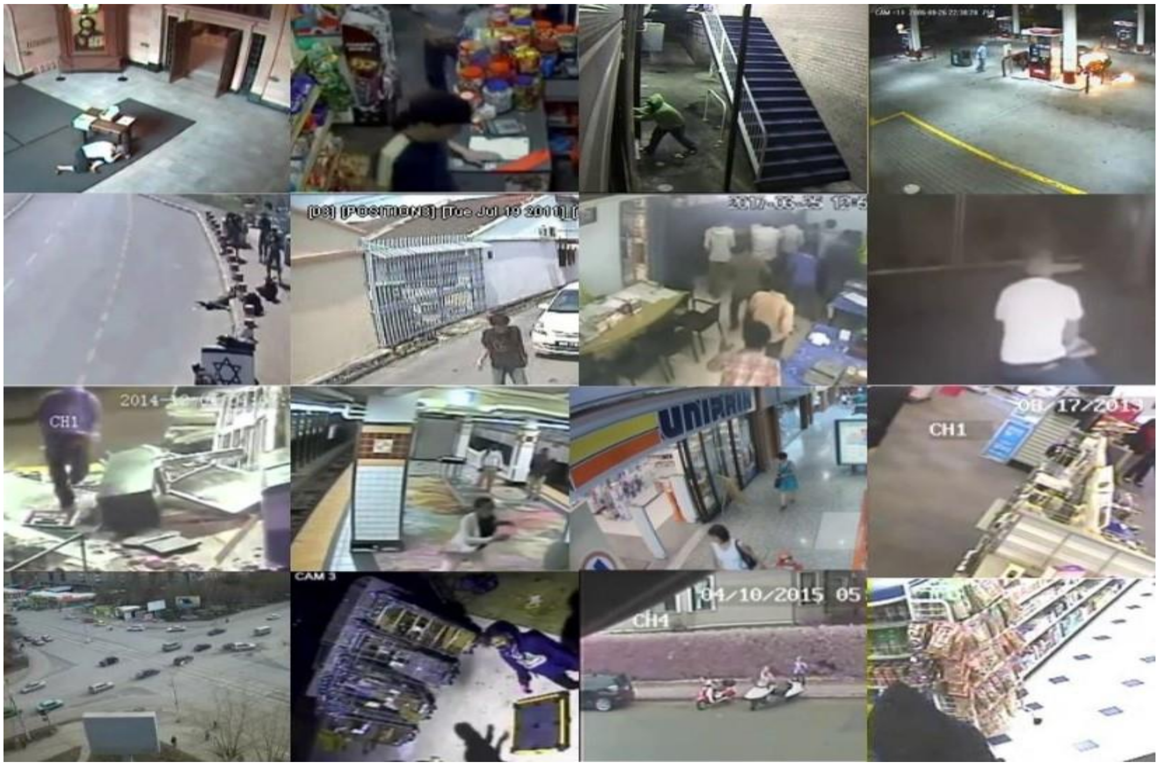

Figure 3 **Extracted key frames samples from UCF-Crime dataset.**

Figure 4

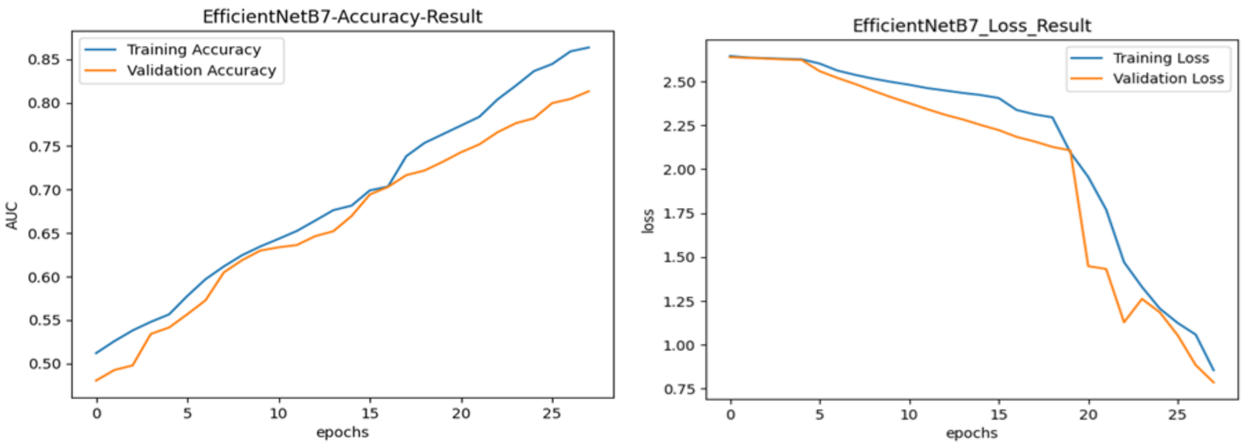

Figure 4 Training and Validation Curves for EfficientNetB7

Figure

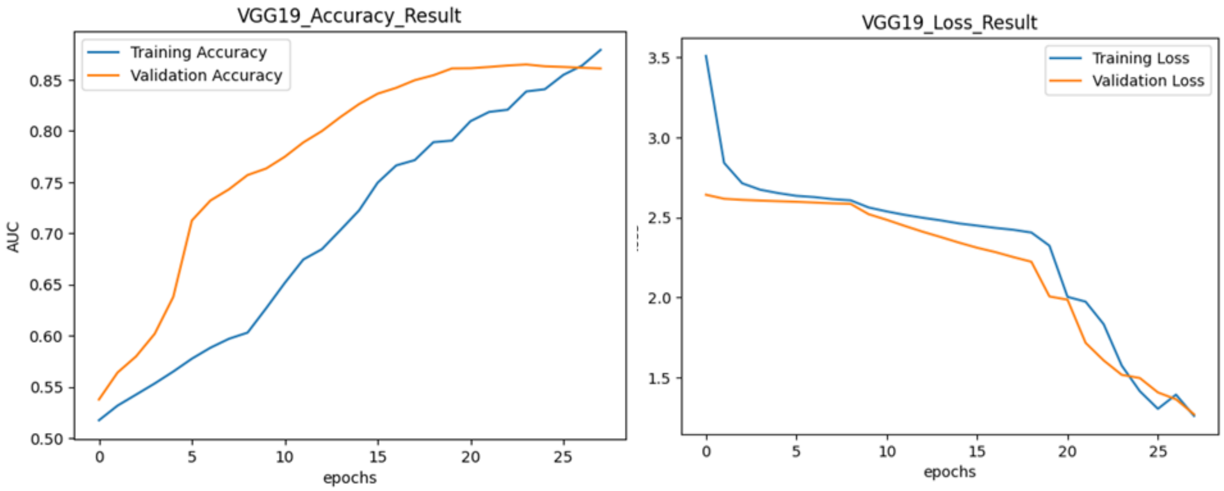

Figure 5 Training and Validation Curves for VGG19

Figure 6

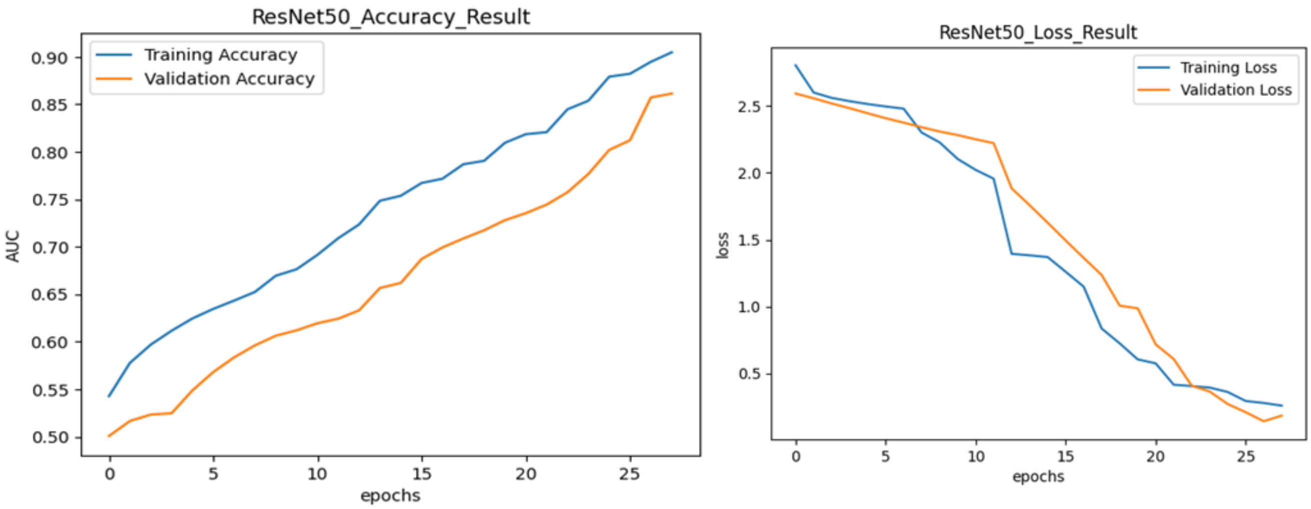

Figure 6 Training and Validation Curves for ResNet50

Figure 7

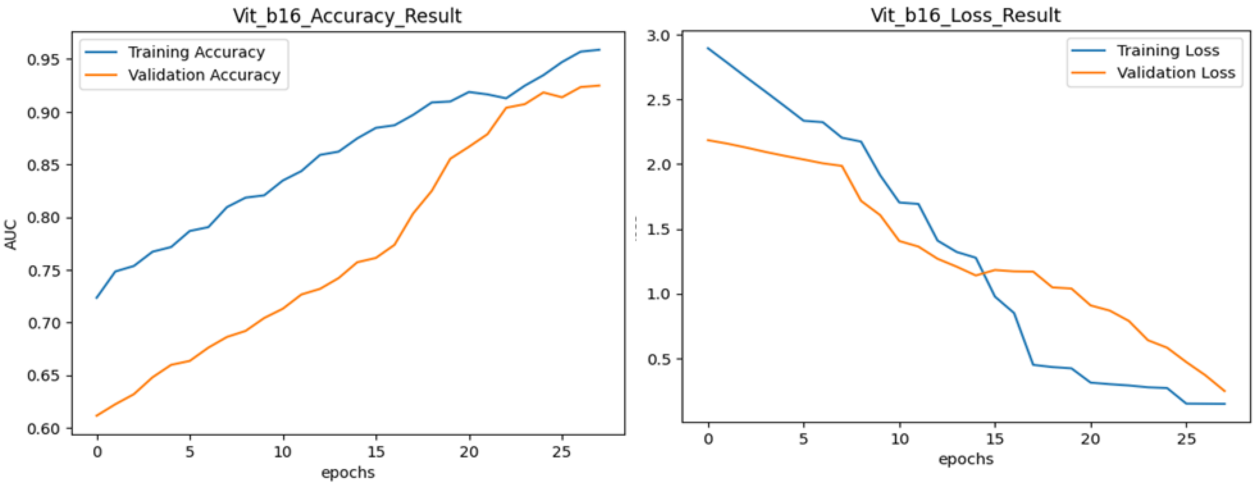

Figure 7 Training and Validation Curves for ViT_b16

Figure 8

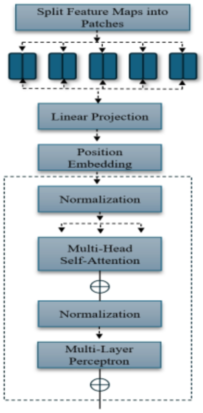

Figure 8 Vit_b16 architecture.
